# Multicenter phase II study of matured dendritic cells pulsed with melanoma cell line lysates in patients with advanced melanoma

**DOI:** 10.1186/1479-5876-8-89

**Published:** 2010-09-27

**Authors:** Antoni Ribas, Luis H Camacho, Sun Min Lee, Evan M Hersh, Charles K Brown, Jon M Richards, Maria Jovie Rodriguez, Victor G Prieto, John A Glaspy, Denise K Oseguera, Jackie Hernandez, Arturo Villanueva, Bartosz Chmielowski, Peggie Mitsky, Nadège Bercovici, Ernesto Wasserman, Didier Landais, Merrick I Ross

**Affiliations:** 1 University of California Los Angeles (UCLA), CA, USA; 2 MD Anderson Cancer Center, Houston, TX, USA; 3 IDM Pharma Inc. (IDM), Irvine, CA, USA; 4 Arizona Cancer Center, Tucson, AZ, USA; 5 Hillman Cancer Center, Pittsburgh, PA, USA; 6 Lutheran General Cancer Care Centre, Park Ridge, IL, USA; 7 AAI Pharma Inc., San Antonio, TX, USA

## Abstract

**Background:**

Several single center studies have provided evidence of immune activation and antitumor activity of therapeutic vaccination with dendritic cells (DC) in patients with metastatic melanoma. The efficacy of this approach in patients with favorable prognosis metastatic melanoma limited to the skin, subcutaneous tissues and lung (stages IIIc, M1a, M1b) was tested in a multicenter two stage phase 2 study with centralized DC manufacturing.

**Methods:**

The vaccine (IDD-3) consisted 8 doses of autologous monocyte-derived matured DC generated in serum-free medium with granulocyte macrophage colony stimulating factor (GM-CSF) and interleukin-13 (IL-13), pulsed with lysates of three allogeneic melanoma cell lines, and matured with interferon gamma. The primary endpoint was antitumor activity.

**Results:**

Among 33 patients who received IDD-3 there was one complete response (CR), two partial responses (PR), and six patients had stable disease (SD) lasting more than eight weeks. The overall prospectively defined tumor growth control rate was 27% (90% confidence interval of 13-46%). IDD-3 administration had minimal toxicity and it resulted in a high frequency of immune activation to immunizing melanoma antigens as assessed by *in vitro *immune monitoring assays.

**Conclusions:**

The administration of matured DC loaded with tumor lysates has significant immunogenicity and antitumor activity in patients with limited metastatic melanoma.

**Clinical trial registration:**

NCT00107159.

## Introduction

Multiple reports have documented the occasional but long lasting responses of metastatic melanoma to several forms of immunotherapy. These approaches include both active tumor-specific immunotherapy with vaccines and non-specific immune stimulants such as cytokines and immune-regulating antibodies [1]. The main theoretical advantage of vaccine approaches resulting in antigen-specific activation is their expected lower toxicity since the stimulation is targeted directly against cancer antigens. *Ex vivo *generated dendritic cells (DCs) are a source of functional antigen presenting cells (APCs) able to present tumor associated antigens (TAAs) to the immune system. The unique ability of DCs to induce and sustain primary immune responses makes them attractive agents in vaccination studies specifically targeting cancer. It was previously shown that DC generated and armed with antigens *ex vivo *can induce effective tumor specific immune responses [2]. In most of the clinical trials reported to date, patients frequently had immune responses while occasional patients had durable clinical responses with limited toxicities [1].

IDD-3 is a cellular therapeutic vaccine consisting of autologous monocyte-derived matured DC, generated from a single apheresis of peripheral blood mononuclear cells (PBMC), cultured in serum-free medium in the presence of the cytokines granulocyte macrophage colony stimulating factor (GM-CSF) and interleukin-13 (IL-13) and pulsed with tumor lysates produced from three allogeneic melanoma cell lines [3,4]. These cell lines were selected because they express proteins that have been identified as common melanoma antigens and they are known to trigger CD8 cytotoxic responses *in vivo *[5,6]. An initial phase I/II study was performed pulsing IDD-3 with just one melanoma cell line lysate (M17) together with hepatitis B surface protein and tetanus toxoid [3]. This pilot study demonstrated the immunogenicity of this vaccine approach and provided early evidence of antitumor activity. One patient with in-transit metastasis had a durable complete response out of 15 patients. A follow up phase I/II study was performed with IDD-3 formulated by pulsing with 3 melanoma cell lines (M44, SKMel28, Colo829), with or without maturation with a bacterial membrane fragment of *Klebsiella pneumoniae *known as FMKp and interferon gamma (IFN-γ) [4]. Twenty-six patients received immature IDD-3 and 23 received mature IDD3. Of the 40 patients eligible for evaluation, 14 showed an immune response against TAAs (Melan-A/MART1, NY-ESO-1, tyrosinase or gp100), with no differences between samples from patients who received immature or matured IDD3. There were no objective tumor responses in this population of patients with more advanced metastatic melanoma.

The present study was undertaken to investigate the antitumor activity of IDD-3 in patients with metastatic melanoma limited to the skin (including in-transit), subcutaneous tissues, lymph nodes or the lung. As suggested by the initial clinical trial with IDD-3 [3], restricting inclusion to patients with limited metastatic disease allows a more adequate patient selection for the testing of this therapeutic vaccine. In this targeted population IDD-3 induced both immune stimulation and had anti-tumor effects.

## Patients and Methods

### Study Design and Conduct

This was a single-arm, two stage, open-label, multi-center phase II study. In the first stage, 12 patients were enrolled, and since a minimum threshold for clinical activity was met, enrollment proceeded to a second stage with up to 38 total patients. A written informed consent, previously approved by the Institutional Review Board at each study site, was obtained from each patient. The study was conducted in accordance with local regulations, the guidelines for Good Clinical Practice (GCP), and the principles of the current version of the Declaration of Helsinki. The study opened to accrual at five US centers and was sponsored by IDM Pharma Inc (Irvine, CA).

### Study Objectives

The primary objective was to assess the clinical activity (as measured by tumor control) following IDD-3 vaccine administration to patients with limited metastatic melanoma. Secondary objectives included the evaluation of immunologic activity of IDD-3 as measured by T-cell responses to melanoma antigens, and to assess the safety of the treatment as measured by the incidence and severity of adverse events.

### Study Population

Patients older than 18 years old with a histologically confirmed primary cutaneous melanoma or melanoma of unknown primary site were eligible. Stage eligibility included non-resected in-transit (Stage IIIb-N2C or stage IIIC-N3), or distant skin, subcutaneous or lymph node (Stage IV-M1a), or pulmonary (Stage IV-M1b) metastases, with serum lactate dehydrogenase (LDH) below 1.5× the institutional upper limit of normal. At least one measurable or evaluable lesion (e.g. small volume cutaneous lesions) was required. There was no restriction on the number of prior therapies, except that patients who had received prior vaccine therapy with one or more melanoma antigens or peptides were excluded. History of autoimmune disease (other than vitiligo), immunodeficiency syndromes (including HIV positive testing), or requirement for chronic systemic immunosuppressive treatment were also excluded.

### IDD-3 Preparation and Administration

A baseline leukapheresis was performed using the COBE Spectra apheresis system (Gambro BCT, Lakewood, CO) according to established procedures for peripheral blood mononuclear cell (PBMC) collection. If needed, up to three leukaphereses could be planned to obtain a target goal of 2 × 10^9 ^PBMC. Within 24 hours of the apheresis, the product was transferred at ambient temperature to the IDM manufacturing facility in Irvine, CA, where DC cells were manufactured in GM-CSF (700 U/mL, Sargramostin, Berlex) and IL-13 (136 ng/mL, Sanofi-Aventis, Labege, France) as previously described [3,4,7]. After a 7 day culture, purified DC were pulsed overnight with 3 melanoma cell line lysates derived from M44 (from F. Jotereau, Nantes, France), COLO829 and SK-MEL28 (both from American Type Culture Collection -ATCC-, Rockville, MD). The Master cell banks and tumor-cell lysates were manufactured and lot release tested by BioReliance Corporation (Rockville, MD). Dendritic cells were then incubated for 6 hours with FMKp (1 μg/mL, Pierre Fabre, St Julien en Genovois, France) and IFN-γ (500 U/mL, Boehringer Ingelheim, Vienne, Austria) to mature the DC. A single IDD-3 dose was made of a sterile suspension of matured and pulsed DC at a concentration of 25 × 10^6 ^cells per mL, cryopreserved in 1 mL of sterile saline with 10% DMSO and 5% human serum albumin. The cell product was stored in labeled vials and cryopreserved in liquid nitrogen. The cryopreserved product was transferred to the clinical site with continuous temperature monitoring. The vaccine was administered within one hour of thawing and reconstituting in 3 mL of sterile saline. Patients were scheduled to receive six IDD-3 immunizations at two-week intervals during the first 10 weeks of treatment, and additional two immunizations at six-week intervals. Each dose of 25 × 10^6 ^cells was administered by injection close to two uninvolved lymph node-bearing regions. For each region, five i.d. injections of 0.1 mL and one s.c. injection of 1.0 mL were performed, giving a total of 3.0 mL per dose. Patients who were felt to have clinical benefit were eligible to continue receiving IDD-3 every eight weeks until all available doses had been administered or the patient experienced disease progression.

### Study Assessments

To evaluate the primary objective of clinical activity, disease assessment was performed at baseline and in weeks 8 and 12. Tumor growth control was expressed as the proportion of patients with a CR or PR maintained for at least four weeks, or SD lasting at least eight weeks, following the Response Evaluation Criteria in Solid Tumors (RECIST) [8]. Lesions in the skin and subcutaneous tissues, evaluable only by physical examination and not detected using imaging studies, were considered measurable if adequately recorded using a camera with a measuring tape or ruler. To evaluate the secondary endpoint of immune responses, blood samples were collected at baseline, prior to the first IDD-3 administration and at various time points thereafter. Cellular immune responses (IFN-γ secretion) to melanoma lysates included in the vaccine, as well as to peptides derived from TAAs, were assessed by ELISPOT assay as previously described [4]. Safety evaluation was also a secondary endpoint, with toxicities evaluated with particular attention paid to injection site reactions (erythema, induration, tenderness, pain, lymph node enlargement), ocular toxicity, fever, autoimmune reactions and vitiligo. All adverse events were graded and documented according to standard criteria (NCI-CTCAE v3.0).

### Sample Procurement and Processing

Blood samples for immune monitoring were collected before vaccination (referred to as w0 sample), during treatment (w4, w8, w12), and during follow-up period (w24 and w48). PBMC were collected through a partial apheresis at baseline and in study week 12, and blood samples were drawn in study weeks 4, 8, 24 and 48. Samples were shipped to the IDM central laboratory (Irvine, CA), where PBMC were obtained after separation over Ficoll gradient centrifugation. Cells were cryopreserved in fetal bovine serum (FBS) containing 10% DMSO, and stored in liquid nitrogen. Before cryopreservation, PBMC suspensions were analyzed for viability, white blood cell content (CD45^+^), and residual presence of granulocytes (CD66b) by flow cytometry.

### *In Vitro *Sensitization

Cryopreserved PBMC samples were thawed, resuspended in AIM V medium completed with 5% human AB serum and 25 mM HEPES (compete Aim V medium), and incubated for 5 minutes at 37°C with 5 U/mL DNase I. Washed cells were incubated overnight. Potential clumps were further eliminated the next day by an optional additional DNase I treatment. PBMC were sensitized to peptide pools *in vitro *during a 14-day culture period. Depending on the number of cells available, 3 to 12 × 10^6 ^viable PBMC were seeded in micro culture plates (2 × 10^5 ^PBMC per well) in complete AIM V medium, with an equivalent number of wells between time points for each pool of peptides. PBMC were stimulated independently with up to 4 pools of peptides derived from single antigen families and restricted by the applicable HLA molecules (Additional file 1, Table S1), with each peptide present in culture at a final concentration of 1 ug/mL. The peptide pools represented the following 4 groups of melanoma tumor antigens: i) gp100 family, ii) tyrosinase and TRP-2 family, iii) MAGE family, and iv) additional miscellaneous tumor antigens AIM-2, Melan-A/MART-1, NY-ESO-1, PRAME, TAG, FGF5, UK, OA1, GPC3, WT1, RNF43, RAGE, and MUM-2 (a detailed list of peptide pools composition is shown in the Additional file 1, Table S1). In addition, a positive control peptide pool made of HLA class I peptides from C Cytomegalovirus, Epstein-Barr Virus, and Flu Virus (CEF peptides, Cellular Technology Ltd., Shaker Heights, OH), and a negative control pool of HIV-derived peptides that cover HLA class I-restricted T cell epitopes were used for the *in vitro *sensitization procedure. Cytokines promoting the expansion of activated T-cells were added to the wells on days 1, 6, and 10 or 11 of the 14 day *in vitro *sensitization culture period. Interleukin (IL)-7 and IL-15 were added at a final concentration of 5 ng/mL and 1 ng/mL, respectively. On days 6 and 10 or 11, the cytokine addition was accompanied by a renewal of half of the culture medium.

### Detection of IFN-γ and CD107a/CD107b by Flow Cytometry

Detection of T-cells specific for tumor antigen epitopes was performed after IVS or directly *ex-vivo *after thawing PBMC samples. Activation of specific T-cells was monitored by production of IFN-γ and exposure to the cell membrane of lysosome-resident CD107a and CD107b proteins in the presence of peptides. Briefly, 10^6 ^cells were incubated for 5 hours with 10 μg/mL of TAA peptides used during the IVS (test sample), an irrelevant HIV peptide pool (negative control), or 25 ng/mL of phorbol 12-myristate 13-acetate (PMA) and 5 μg/mL ionomycin (positive control). Cells sensitized with the viral CEF pool were incubated with the negative control HIV pool, 2 μg/mL of the positive control CEF peptide pool, or PMA/ionomycin as non-specific positive control. The incubation was performed in the presence of anti-CD107a and anti-CD107b antibodies. Brefeldin A (10 μg/mL) and Golgi stop (monensin, 6 μg/mL) were added 1 hour after peptide stimulation. Cells were then stained with anti-CD8-PE and anti-CD3-PerCP antibodies, fixed and permeabilized with the Intrastain kit, and stained for intracellular IFN-α with an anti-IFN-γ-APC antibody. Cells were resuspended and stored for up to 4 days in PBS 1% Cytofix before being analyzed on a FACSCalibur flow cytometer. Anti-TAA specific CD8^+ ^cells were defined as cells producing IFN-γ (with or without CD107 expression) after stimulation with TAA-peptide pools. The CD8^+ ^T cell population was defined by gating on lymphocytes according to size and structure (FSC/SSC), followed by gating on CD8^+ ^CD3^+ ^lymphocytes. An average of 80,000 ± 40,000 CD8^+ ^events were collected from each sample. Data shown in this report are expressed as the proportion of net TAA-specific CD8^+ ^events for 10^5 ^total CD8^+ ^cells.

### Detection of Melan A-specific T Cells with MHC tetramers

When sufficient cells from HLA-A*0201 positive subjects were available, T cells (10^6 ^cells) were stained with Melan A/MART-1 tetramers or neg-tetramers as control (all from Beckman Coulter), and with anti-CD8-FITC (BD Pharmingen) as recommended by the manufacturers. After washing, cells were stained with TOPRO3 (Molecular probe) to exclude dead cells and were analyzed by flow cytometry.

### Sample Size Determination and Statistical Analysis

The clinical trial followed a Simon two-stage optimal design [9] with an assumed tumor growth control (objective responses by RECIST plus SD beyond 8 weeks) rate of 20%, a null response rate of 5%, a type one error of 0.10, and a type two error of 0.10. As such, continuation to the second stage required one patient out of the first 12 recruited to demonstrate positive tumor growth control during the first 12 weeks of treatment. After completing the second stage, if four or more out of 37 patients were observed with tumor growth control the trial was deemed positive. The probability of early termination due to an unacceptably low response (i.e., the null response rate is true) was 54%. Assessment of the secondary objective of immunological activity was determined by determining the proportion of patients showing an induction or increase in immune response to melanoma lysates or TAAs following treatment. Patients evaluable for immune response were those eligible patients who had received at least two doses of IDD-3 vaccines, had a valid baseline immune response assessment and provided at least one post-vaccination blood sample. A patient was considered to have a positive immune response to treatment if there was a two-fold or greater increase in the lysate- or peptide-specific T-cell responses or antibody titer at any post-vaccination time point compared to the pre-vaccination sample. For each IVS condition (for example, stimulation with peptide pool 1), the number of IFN-γ positive events (and negative events) on CD8^+ ^CD3^+ ^lymphocytes were analyzed for stimulations with HIV peptides, TAA peptides or PMA/ionomycin. A test sample was considered positive and T cells specific for a peptide pool were considered detectable if the following conditions were met. First, the positive control (PMA/ionomycin) displayed a significantly higher number of IFN-γ positive CD8^+ ^CD3^+ ^cells than the negative control HIV pool by the Chi-square test, provided that the Chi square test was applicable between test sample and negative control samples since the number of events was sufficient. Second, the test sample displayed a significantly higher number of IFN-γ positive CD8^+ ^CD3^+ ^cells than the negative control by the Chi-square test, provided that the number of IFN-γ positive CD8^+ ^CD3^+ ^cells in the test sample was at least twice that in the negative control and the difference of these two numbers was at least 0.1% of the total CD8^+ ^CD3^+ ^population.

## Results

### Study Patients

Between February, 2005 and August, 2006, a total of 45 patients were assessed for study entry. Seven patients did not meet the eligibility criteria after screening tests. Patients were recruited from five centers in the USA; the majority of recruitment (82% of patients) occurred just at two centers. The demographic characteristics of all 38 patients that met the eligibility criteria are presented in Table 1. Five of these patients (13%) did not receive IDD-3, three due to rapid disease progression before the start of treatment and two because of unsuccessful vaccine manufacture (Table 2). All patients had histologically confirmed stage III or IV melanoma, the majority (68%) with metastases confined to the skin (M1a) and/or the lung (M1b). One patient (#093-120) was included with a protocol waiver approved by the local IRB after being found to have low volume liver metastases (M1c) at the baseline scans. All patients had normal serum LDH at study entry. Twenty patients (53%) had received prior immunotherapy and 17 patients (45%) had received prior chemotherapy.

**Table 1:** Patient Characteristics

Characteristic	Number	%
Number of patients	38	100%
Sex		
Male	22	58%
Female	16	42%
Age (years)		
Median	60	
Range	27-85	
ECOG PS at inclusion		
0	28	74%
1	10	26%
2	0	0%
3	0	0%
ECOG PS at treatment initiation		
0	23	70%
1	8	24%
2	1	3%
3	1	3%
Staging at study entry		
IIIB - N2c	5	13%
IIIC - N3	6	16%
IV - M1a	16	42%
IV - M1b	10	26%
IV - M1c	1	3%
Number of organs involved at baseline		
Median (range)	1 (1-4)	
1	27	71%
2	8	21%
> 2	3	8%
Organs involved		
Local recurrence	2	5%
Skin metastasis	18	47%
Lymph node	11	29%
Lung metastasis	17	45%
Prior therapies		
Prior radiotherapy	7	18%
Prior immunotherapy	20	53%
Prior immunotherapy and chemotherapy	7	18%
Prior systemic chemotherapy	17	45%
Number of prior chemotherapy lines; Median (range)	1 (0-4)	
Prior Isolated Limb Perfusion	6	16%

Abbreviations: ECOG-PS: Eastern Cooperative Oncology Group Performance Status;

**Table 2:** IDD-3 Administration

	N
Number of treated patients	33
Total number of doses administered	243
Number of doses administered per patient:	
Median (range)	7 (2-14)
Number of patients with ≥ 6 doses (w0 to w10)	26 (79%)
Number of patients with ≥ 8 doses (w0 to w22)	13 (39%)
Number of patients discontinuing prior to treatment initiation	5 (13%)
Discontinued due to:	
IDD-3 manufacture failure	2 (5%)
Early disease progression	3 (8%)

### IDD-3 Vaccine Manufacture

Three patients required more than one leukapheresis in order to obtain enough cells for vaccine manufacture (target goal of >2 × 10^9 ^PBMC); one of them required three procedures. For the other 35 patients, a single leukapheresis was sufficient. As described above, the leukapheresis products from two patients did not yield IDD-3 vaccines that met the lot release criteria. Therefore, IDD-3 was not administered to these two patients, resulting in 33 patients receiving at least two doses of IDD-3 (Table 2). The median number of IDD-3 administrations per patient was seven (range 2-14). Most patients (79%) received at least six vaccinations, with over one third (39%) completing the planned eight IDD-3 administrations.

### Toxicity and Adverse Events

There was a single serious adverse event leading to a study discontinuation in a patient who developed grade 3 macular degeneration that was considered possibly related to the experimental agent by the study investigators. Otherwise, IDD-3 administration was very well tolerated in this patient population. The most frequent- treatment-related adverse events were mild (grade 1-2) and included injection site reactions (52% of patients), fatigue (36%), myalgias (30%) and headache (9%).

### Clinical Efficacy

One patient had a confirmed CR, two patients had a PR, and six patients (18%) had SD lasting more than eight weeks as their best response, for an overall tumor growth control rate (objective tumor response plus SD beyond 8 weeks) of 27% (90% confidence interval of 13 to 46%, Table 3). This rate is beyond the prospectively specified target tumor growth control rate of 20%.

**Table 3:** Response Assessment

Response	N	%
Number of patients	33	100%
Number of patients with:		
CR	1	3%
PR	2	6%
SD > 8 weeks	6	18%
SD ≤ 8 weeks	9	27%
PD	15	45%
Tumor Growth Control (90% CI)	9	27% (13% - 46%)
Overall Response Rate (90% CI)	3	9% (3% - 22%)

Abbreviations: CR: complete response; PR: partial response; SD: stable disease; PD: progressive disease.

**Figure 1:**
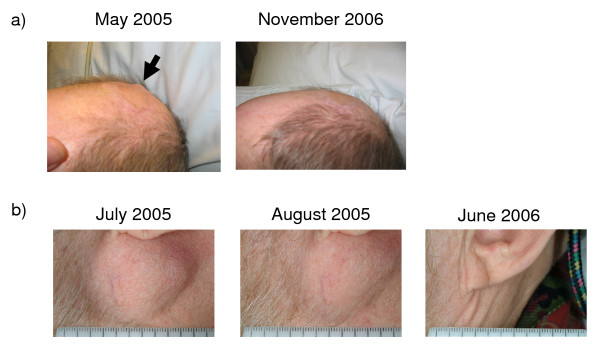
**Antitumor response in patient 093-020**. a) Evolution of subcutaneous scalp metastases. b) Evolution of subauricular nodal metastases.

**Figure 2:**
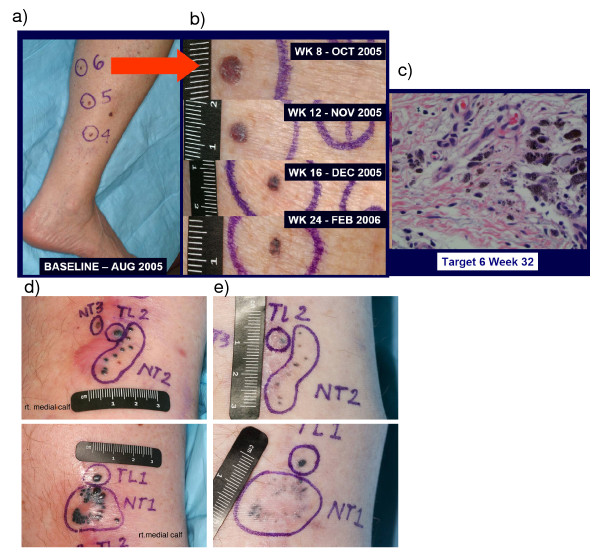
**Antitumor response and pathologic analysis in patients 095-050 (a-c) and patient 095-200**. a) Baseline picture of skin metastasis in the right lower extremity. b) Close-up pictures of the evolution of target lesion 6 in patient 095-050. c) H&E image of the pathologic analysis of a residually pigmented skin lesion from target lesion 6 on week 32, demonstrating melanophages and no evidence of active melanoma. d) Baseline lesions in patient 095-200. e) The lesions at week 40 were smaller but retained pigmentation. No viable tumor was detected upon biopsy.

Table 4 provides details of the patients with tumor growth control. Patient #093-020 had scalp and bulky cervical lymph node metastases progressing after 4 prior surgical resections and had not received prior systemic therapy for metastatic disease. The patient received a total of 14 administrations of IDD-3 leading to a slowly evolving CR (Figure 1). This patient died of unrelated causes 30 months after enrollment, and was melanoma-free at autopsy. Patient #095-050 had in-transit metastases in the right leg and had undergone multiple prior surgical resections and two rounds of isolated limb perfusion (one with melphalan and another one with melphalan and actimomycin-D). The patient had evidence of progressive disease 8 months after the second isolated limb perfusion and then received a total of 12 vaccinations resulting in a durable PR (Figure 2). The week 12 assessment showed an increase in the size of these lesions, but further assessments showed a significant regression of cutaneous lesions (concomitant changes in pigmentation and nodularity) with continued dosing. Resection of two nodules at weeks 24 and 36 showed no evidence of viable melanoma; it was consistent with a complete pathological CR at these metastatic sites (Figure 2a, b, c). The patient was progression-free at the last follow-up, 33+ months after clinical trial initiation. Patient 095-200 had skin metastasis progressing shortly after receiving adjuvant interferon. The patient received a total of 8 vaccinations and achieved a PR (Figure 2d, e), being free of progression for 35 months until a tumor relapse was documented in soft tissues.

**Table 4:** Summary of characteristics of patients showing clinical response

Patient #	Gender/Age	Stage	Prior treatment	**Best Response**^**1**^	Duration (months)
# 093-020	Male/60 y	M1a	Surgery	CR	30
# 095-050	Female/70 y	IIIc	Surgery-ILP	PR	33+
# 095-200	Male/46 y	M1a	Surgery/Interferon	PR	35+
# 095-020	Female/44 y	M1b	Surgery-ILP	SD/NED	30+
# 095-060	Female/69 y	M1b	Surgery	SD/NED	22+
# 093-170	Female/84 y	M1a	Surgery	SD	13
# 095-080	Male/55 y	M1a	Surgery-Biotherapy	SD	10
# 095-190	Female/54 y	M1a	Surgery/Cilengitide	SD	10
# 096-020	Female/58 y	M1b	Surgery-RT-Chemo-Biotherapy	SD	5

Abbreviations: RT: radiotherapy; ILP: isolated limb perfusion; CR: complete response; PR: partial response; SD: stable disease; NED: no evidence of disease.

Two additional patients who did not meet the criteria of objective response by RECIST may have benefited from study participation. Patient #095-020 with skin and nodal metastases in the right leg previously treated with isolated limb perfusion with melphalan entered the study after the development of lung metastases for which he had not received prior active systemic therapy. The patient received a total of 12 vaccinations with a best response of SD; the therapy resulted in a significant arrest of growth of the lung metastasis. The patient underwent surgical resection of all active sites of disease at 13 months after initiating vaccine administration. Several of the removed in transit lesions showed no evidence of viable melanoma. After surgery the patient had no evidence of disease (NED), which persisted at last follow-up at 24+ months. Patient 095-060 was enrolled with lung metastases of a mucosal melanoma of nasal primary, without previous systemic treatment. The patient received a total of 10 IDD-3 administrations, resulting in SD. After surgical resection of lung metastases, the patient was rendered NED. The patient has had no disease progression at 30+ months of follow up. In addition, four patients (#093-170, #095-080, #095-190 and #096-020) had stable skin and/or nodal metastasis for 5 to 13 months before disease progression.

### Immune Response

**Figure 3:**
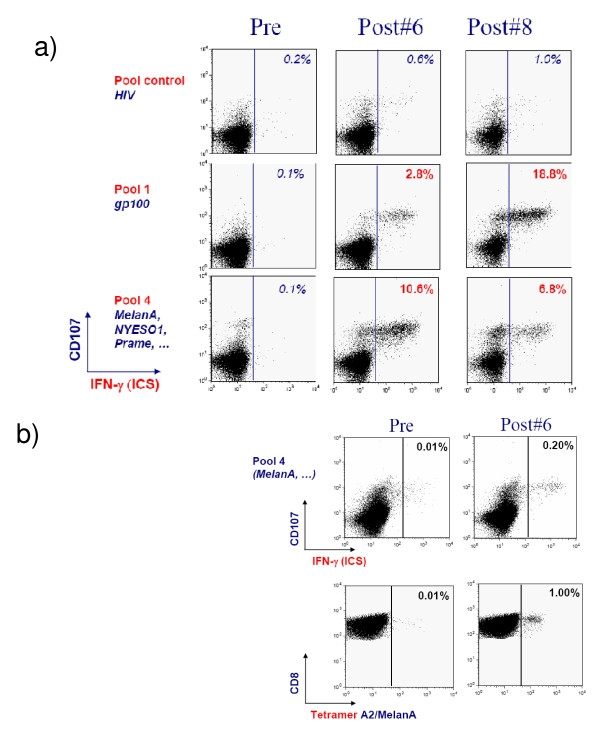
**Examples of flow cytometric analysis with double staining for CD107a (y axis) and interferon gamma (x axis) by in vitro sensitized (IVS) peripheral blood mononuclear cells (PBMC) from two patients with an objective response to IDD-3 vaccination**. a) Samples from patient 093-020. b) Samples from patient 095-050. Detection of melanA-specific CD8 T cells with tetramers is also shown for this patient.

Twenty-nine of the 33 patients (76%) who received IDD-3 vaccine treatment were evaluable for an immune response because they had adequately cryopreserved PBMC samples from before and at least one time point after initiating IDD-3 administration. Two examples of detection of TAA-specific CD8^+ ^T cells pre- and post-vaccination are shown in Figure 3. Among 29 patients assessed for immune responses, 26 patients (90%) had detectable TAA-specific CD8+ T cells in peripheral blood. We had prospectively defined an increased immune response to treatment if patients showed a 2-fold increase over baseline at one (or more) time points post-treatment. The data are summarized in the Additional file 2, Table S2. Among 26 patients with detectable TAA-specific CD8^+ ^T cells, we could discriminate 3 groups of patients: a) patients with boosted/induced immune responses post-treatment to a single pool or multiple pools of TAA-derived peptides (n = 19, 66%); b) patients with stable immune response to one (or more) pool (n = 3); and c) patients with decreased immune response to one (or more) pool (n = 4). Tetramer staining was investigated in one HLA-A*0201 positive patient who had sufficient number of cryopreserved cells pre- and post-vaccination. We could show that Melan A-specific CD8^+ ^T cells were indeed detectable in the post-vaccination sample (Figure 3b).

## Discussion

There have been over 100 clinical trials testing the antitumor activity of DC vaccines over the past 10 years [10]. Most have been small pilot studies in single institutions, frequently without pre-defined parameters for quality control of the DC manufacturing procedures. The methods for DC generation, maturation and administration to patients have varied widely with seemingly little impact on the low response rates of this approach. The great majority of these studies included vaccination with DCs generated by one week of *ex vivo *culture in the presence of GM-CSF and IL-4, with or without additional maturation. Overall, DC vaccines have resulted in very low response rates [10]. One large multicenter randomized clinical trial tested the administration of matured dendritic cells compared to DTIC in patients with metastatic melanoma [11]. In this work, DC generated in GM-CSF and IL-4 were matured with TNF-α, IL-1β, IL-6 and prostaglandin E2 (PgE2), pulsed with a mixture of 20 MHC class I and II-binding peptides and administered subcutaneously. Disappointingly, the DC arm had a response rate that was statistically inferior to DTIC. The current study differs from this experience in the DC manufacturing process and its administration to patients with relatively limited metastatic disease.

It is highly likely that patient selection has a major role in the results of our study and it compares favorably to several prior DC-based approaches. Most of the patients had metastatic disease localized in skin and soft tissues, including in-transit lesions. Three patients achieved objective and durable tumor responses, including one patient who developed a PR after early evidence of disease progression, a phenomenon that has been documented with other immunotherapeutic approaches for this disease [12,13]. The three patients with an objective response had undergone prior surgery with the addition of either isolated limb perfusion with chemotherapy or systemic adjuvant therapy with interferon, but had not received prior systemic chemotherapy. In this small study it is unclear if the prior therapy was a major determinant for response. It is more likely that the disease stage, with limited skin and nodal metastasis, may be a more important determinant of response than limited prior systemic cytotoxic therapy. As the number of observed objective responses and SD beyond 8 weeks exceeds the minimum requirement defined in the pre-specified study hypothesis, we concluded that further evaluation of this regimen in the same population of patients with metastatic melanoma M1a and M1b (skin, nodal and lung metastases) is merited.

However, the current study failed to provide a clear correlation between results of immune monitoring and clinical benefit in terms of tumor responses. This lack of correlation of immune parameters and response may relate to the likely low sensitivity of the detection of immune responses to uncharacterized antigens in the tumor lysates used to pulse DCs. It is also likely that analysis of T cell responses in peripheral blood may not fully recapitulate events in tumors as has been described for therapy with anti-CTLA4 antibodies [14]. Finally, the detection of T cells stimulated by a vaccine may not have a correlation with tumor regression, since some cancer cells may not be adequate targets for T cells even when a robust T cell response has been induced by the vaccine. For example, low antigen expression, low MHC expression or other antigen processing and presenting molecule alteration, and insensitivity to the pro-apoptotic signals from T cells would all lead to a disconnect between the results of an immune monitoring assay in peripheral blood and tumor responses [15].

The results of the current study should be interpreted in the context of other recently reported vaccine and immunotherapy approaches for cancer. There have been two large randomized trials suggesting that adjuvant therapy with cancer vaccines may be detrimental to patients with surgically treated melanoma. One approach used a mixture of melanoma tumor cell lysates administered with BCG and the other used a ganglioside vaccine administered with a strong KLH adjuvant [16]. These results and the negative experience with DC vaccines compared to standard chemotherapy [11], have reduced enthusiasm for immunotherapy base on prior small, uncontrolled trials. However, the recent report that treatment with the immune modulating antibody anti-CTLA4 antibody ipilimumab prolonged survival compared to a gp100 vaccine in patients with previously treated metastatic melanoma should invigorate the field of immunotherapy [17]. CTLA4 blocking monoclonal antibodies induce durable objective responses in some patients with melanoma mediated by T cell infiltrates in tumors [14], attesting to the immune nature of their benefit. However, CTLA4 blockade is a mode of non-specific immune activation by abrogating a negative regulatory mechanism of the immune system. This is mechanistically quite different from inducing a T cell response to cancer antigens using a vaccine. One option is to combine both approaches. In a pilot phase I trial of the anti-CTLA4 antibody tremelimumab plus a MART-1 peptide-pulsed DC vaccine, objective and durable tumor responses were seen at higher level than what would have been expected with either approach alone [18]. However, this pilot study was too small to provide firm data on the benefits of this combination. Further exploration of such combinations of a vaccine and immune modulating antibodies or cytokines is warranted. It ideally should build upon a vaccine like the one presented in this study, with single agent activity in terms of tumor responses. At the same time we must use caution when we plan these type studies since the combination treatments may result in worsening of the response rate as it was seen in the aforementioned study of ipilimumab and gp100 [17].

In conclusion, we report the positive results of a DC-based vaccine study in patients with metastatic melanoma selected for good prognostic features. This multi-center study used a centralized GMP facility that processed patient's own blood cells to generate tumor lysate-loaded and matured DC vaccines. Despite the encouraging data of the clinical trial reported herein, with objective responses without significant toxicities, the company that conducted this multicenter study (IDM) could not proceed with this line of research. A multicenter, randomized phase II clinical trial (clinical trial registration number NCT00107159) was discontinued due to lack of funds. Regardless, the study presented herein demonstrates the feasibility of a matured DC vaccination approach in a multi-center study and confirms the low but reproducible durable response rate achievable by this mode of active cellular immunotherapy.

## Competing interests

While this research was being conducted Sun Min Lee, Peggie Mitsky, Nadège Bercovici and Didier Landais were employees of IDM Pharma, the company that developed IDD3. In addition, Ernesto Wasserman was an employee of AAI Pharma Inc., San Antonio, TX, which was contacted by IDM Pharma for the conduct of this study. The other investigators have no competing interests.

## Authors' contributions

SML, PM, NB and DL designed the study. AR, LHC, EMH, CKB, JMR, JAG, DKO, BC and MIR treated patients within this protocol. VGP performed pathological analysis of samples. MJR, JH, and AV performed study coordination and data management. NB performed immunological analysis of samples. PM, EW and DL were responsible for procedures to generate the IDD3 vaccines and conducted the study for the sponsor. AR, LHC, EMH, BC, NB, EW and DL wrote the manuscript. All authors approved the final manuscript.

## Supplementary Material

Additional file 1 **Peptide pools**. Table detailing the protein location and amino acid sequence of peptides used in the different peptide pools used for immunological analysis of patient-derived samples. Click here for file

Additional file 2 **Summary of immune responses to IDD3**. Table detailing the study subjects, their clinical response and their immune response based on reactivity to tumor antigens by intracellular cytokine staining by flow cytometry. Click here for file
