# Supplementary material for: Multicenter phase II study of matured dendritic cells pulsed with melanoma cell line lysates in patients with advanced melanoma
Source: J Transl Med. 2010 Sep 27;8:89. doi: 10.1186/1479-5876-8-89 (PMC2954849; doi:10.1186/1479-5876-8-89)
Supplement: Additional file 1 — Peptide pools. Table detailing the protein location and amino acid sequence of peptides used in the different peptide pools used for immunological analysis of patient-derived samples. [file 1479-5876-8-89-S1.DOC]

**Additional Table 1.**

| **Pool #** | **Antigen** | **HLA Restriction** | **Sequence and Position** | **Peptide Name** |
| --- | --- | --- | --- | --- |
| 1 | gp100 | A2 | IMDQVPFSV 209-217 | gp100 A2b |
|  |  |  | KTWGQYWQV 154-162 | gp100 A2c |
|  |  |  | YLEPGPVTA 280-288 | gp100 A2d |
|  |  |  | VLAQVVLQL 286-294 | gp100 A2e |
|  |  |  | LLDGTATLRL 457-466 | gp100 A2f |
|  |  |  | VLYRYGSFSV 476-485 | gp100 A2g |
|  |  |  | RLMKQDFSV 619-627 | gp100 A2h |
|  |  |  | RLPRIFCSC 639-647 | gp100 A2i |
|  |  | A2/A3 | ALLAVGATK 17-25 | gp100 A2a/A3c |
|  |  | A3 | AVVLASLIYR 608-617 | gp100 A3a |
|  |  |  | LIYRRRLMK 614-622 | gp100 A3b |
|  |  |  | IALNFPGSQK 86-95 | gp100 A3e |
|  |  | A3/A11 | ALNFPGSQK 87-95 | gp100 A3d/A11 |
|  |  | A24 | VYFFLPDHL intron 4 | gp100 A24 |
|  |  | B7 | SSPGCQPPA 529-537 | gp100 B7a |
|  |  | A1/B35/B7 | LPHSSSHWL 630-638 | gp100 A1/B35/B7 |
|  |  | B35 | VPLDCVLYRY 471-480 | gp100 B35a |
| 2 | Tyr | A1 | KSDICTDEY 243-251 (244S) | Tyr A1a |
|  |  |  | DSDPDSFQDY 454-463 | Tyr A1b |
|  |  |  | SSDYVIPIGTY 146-156 | Tyr A1c |
|  |  |  | DAEKSDICTDEY 240-251 | Tyr A1d |
|  |  | A2 | YMDGTMSQV 369-377 | Tyr A2a |
|  |  |  | MLLAVLYCL 1-9 | Tyr A2b |
|  |  |  | CLLWSFQTSA 8-17 | Tyr A2c |
|  |  | A3/A11 | YMVPFIPLYR 425-434 | Tyr A3/A11a |
|  |  | A11 | QTSAGHFPR 14-22 | Tyr A11b |
|  |  | A24 | AFLPWHRLF 206-214 | Tyr A24 |
|  |  | B7 | LPWHRLFLL 208-216 | Tyr B7 |
|  |  | B35 | LPSSADVEF 312-320 | Tyr B35a |
|  |  |  | TPRLPSSADVEF 309-320 | Tyr B35b |
|  |  | B44 | SEIWRDIDF 192-200 | Tyr B44 |
|  | TRP-2 | A1 | VYDFFVWLHY 181-190 | TRP2 A1 |
|  |  | A2 | SVYDFFVWL 180-188 | TRP2 A2a |
|  |  |  | FLWLHYYSL 185-193 | TRP2 A2b |
|  |  |  | SLDDYNHLV 288-296 | TRP2 A2c |
|  |  |  | TLDSQVMSL 360-368 | TRP2 A2d |
|  |  |  | ATTNILEHY 403-411 | TRP2 A2e |
|  |  | A3 | LLGPGRPYR 197-205 | TRP2 A31 |
| 3 | MAGE | A2 | KVLEYVIKV 278-286 | MAGE-1 A2 |
|  |  |  | LLHFLLLKL 116-124 | MAGE-2 A2a |
|  |  |  | YLQLVFGIEV 157-166 | MAGE-2 A2b |
|  |  |  | LLFGLALIEV 191-200 | MAGE-C2/A2a |
|  |  |  | ALKDVEERV 336-344 | MAGE-C2/A2b |
|  |  |  | KVAELVHFL 112-120 | MAGE-3/A2 |
|  |  |  | ALSVMGVYV 223-231 | MAGE-9/A2 |
|  |  |  | GVYDGREHTV 230-239 | MAGE-4/A2 |
|  |  |  | GLYDGMEHL 254-262 | MAGE-10/A2 |
|  |  | A3 | SLFRAVITK 96-104 | MAGE-1/A3 |
|  |  | A24 | NYKCRFPEI 135-143 | MAGE-1/A24 |
|  |  |  | EYLQLVFGI 156-164 | MAGE-2/A24 |
|  |  |  | IMPKAGLLI 195-203 | MAGE-3/A24a |
|  |  |  | TFPDLESEF 97-105 | MAGE-3/A24b |
|  |  |  | NYKRCFPVI 143-151 | MAGE-4/A24 |
|  |  | B7 | RVRFFFPSL 289-297 | MAGE-1 B7 |
|  |  | A1 | EADPTGHSY 161-169 | MAGE-1 A1 |
|  |  | A1/B35 | EVDPIGHLY 168-176 | MAGE-3 A1/B35 |
|  |  | B35 | EVDPIGHVY 168-176 175V | MAGE-6 B35 |
|  |  | B44 | MEVDPIGHLY 167-176 | MAGE-3 B44 |
| 4 | NY-ESO-1 | A2 | SLLMWITQA 157-165 (165A) | NY-ESO-1 A2a |
|  |  |  | MLMAQEALAFL ORF2 (1-11) | NY-ESO-1 A2b |
|  |  | A3 | AAQERRVPR 9-19 | NY-ESO-1 A31 |
|  |  | A24 | LLMWITQCF 158-166 | NY-ESO-1 A24 |
|  |  | B7 | APRGVRMAV ORF2 (46-54) | NY-ESO-1 B7 |
|  |  | B35/B51 | MPFATPMEA 94-102 | NY-ESO-1 B35/B51 |
|  | Melan-A | A2 | ELAGIGILTV 26-35 (27L) | Melan-A 27L A2 |
|  |  |  | ILTVILGVL 32-40 | Melan-A A2b |
|  |  | B35 | EAAGIGILTY 26-35 (35Y) | Melan-A B35 |
|  | AIM-2 | A1 | RSDSGQQARY intron | AIM-2 A1 |
|  | PRAME | A2 | VLDGLDVLL 100-108 | PRAME A2a |
|  |  |  | ALYVDSLFFL 300-309 | PRAME A2b |
|  |  |  | SLLQHLIGL 425-433 | PRAME A2c |
|  |  | A24 | LYVDSLFFL 301-309 | PRAME A24 |
|  | TAG | A3 | RLSNRLLLR | TAG A3 |
|  | FGF5 | A3 | NTYASPRFK 172-176 | FGF5 A3 |
|  | UK | A3 | SQNFPGSQK | UK A3 |
|  | OA1 | A24 | LYSACFWWL 126-134 | OA1 A24 |
|  | GPC3 | A24 | EYILSLEEL 298-306 | GPC3 A24 |
|  | WT1 | A24/B7 | CMTWNQMNL 235-243 | WT1 A24/B7 |
|  | RNF43 | A24 | NSQPVWLCL 721-729 | RNF43 A24 |
|  | RAGE-1 | B7 | SPSSNRIRNT 44136 | RAGE-1 B7 |
|  | MUM-2 | B44 | SELFRSGLDSY 123-133 | MUM-2 B44 |
