# Supplementary material for: Multicenter phase II study of matured dendritic cells pulsed with melanoma cell line lysates in patients with advanced melanoma
Source: J Transl Med. 2010 Sep 27;8:89. doi: 10.1186/1479-5876-8-89 (PMC2954849; doi:10.1186/1479-5876-8-89)
Supplement: Additional file 2 — Summary of immune responses to IDD3. Table detailing the study subjects, their clinical response and their immune response based on reactivity to tumor antigens by intracellular cytokine staining by flow cytometry. [file 1479-5876-8-89-S2.DOC]

**Additional Table 2.** Summary of immune responses to IDD3.

|  | **Immune Responses**  **to Vaccination** | **Patient ID** | **HLA subtypes** | **Clinical Status** | **gp 100** | **Tyr/TRP** | **Mage** | **Miscel** |
| --- | --- | --- | --- | --- | --- | --- | --- | --- |
| **1** | **Boosted/**  **Induced**  **Responses**  **(n=19)** | 9512 JMJ | A02, A25, B39, B44 | PD |  |  |  |  |
| **2** |  | 9511 ELO | A02 A32 B1401 B51 | PD |  |  |  |  |
| **3** |  | **9302 TR** | A01 A201 B0702 B0801 | **CR** |  |  |  |  |
| **4** |  | 9513 CCR | A0201, A68, B1402, B15 | PD |  |  |  |  |
| **5** |  | 9308 WC | A01 A02 B44 B58 | PD |  |  |  |  |
| **6** |  | 9509 KJS | A11 A29 B35 B44 | PD |  |  |  |  |
| **7** |  | **9520 SBA** | NA | **PR** |  |  |  |  |
| **8** |  | **9519 PAK** | NA | **SD** |  |  |  |  |
| **9** |  | 9518 SNS |  | PD |  |  |  |  |
| **10** |  | 9307 ID | A23 A68 B14 B41 | PD |  |  |  |  |
| **11** |  | 9306 JO | A03 A26 B07 B44 | PD |  |  |  |  |
| **12** |  | 9504 BRC | A03, B07, B15 | PD |  |  |  |  |
| **13** |  | **9506 SMB** | A03, A23, B07, B15 | **SD** |  |  |  |  |
| **14** |  | 9316 PG | A03, A24, B39, B60 | PD |  |  |  |  |
| **15** |  | 9304 JK | A1, A25 | PD |  |  |  |  |
| **16** |  | **9505 TLK** | A1, A2, B7, B3906 | **PR** |  |  |  |  |
| **17** |  | 9516 RJM | A2, A24, B18, B44 | PD |  |  |  |  |
| **18** |  | 9310 TK | A24, A31, B07, B44 | PD |  |  |  |  |
| **19** |  | **9317 MF** |  | **SD** |  |  |  |  |
| **20** | **Stable**  **Responses**  **(n=3)** | 9305 EV | A1, A2 | PD |  |  |  |  |
| **21** |  | **9508 LDF** | A2, A11 B44 B51 | **SD** |  |  |  |  |
| **22** |  | 9312 VH | A29, B08, B45 | PD |  |  |  |  |
| **23** | **Decreased**  **Responses**  **(n=4)** | **9602 NJL** | A02, A03, B15, B18 | **SD** |  |  |  |  |
| **24** |  | 9311 EA | A0206, A31, B35, B39 | PD |  |  |  |  |
| **25** |  | 9223 GJC | A0201, A0202, B41, B4402 | PD |  |  |  |  |
| **26** |  | **9502 SAT** | A1, A31, B7, B18 | **SD** |  |  |  |  |
| **27** | **No TAA-**  **specific CTL**  **(n=3)** | 9501 RDF | A28, A32, B27, B51 | PD |  |  |  |  |
| **28** |  | 9503 RMM | A24, A29 B27 B44 | PD |  |  |  |  |
| **29** |  | 9517 SND | A1, A3, B7, B8 | PD |  |  |  |  |
|  |  |  |  |  |  |  |  |  |
|  | No TAA-specific CD8 cells detected. | | | | | | | |
|  | Stable TAA-specific CD 8* cells (pre-existing response and stable response detected post-vacc) | | | | | | | |
|  | Increase in frequency of TAA-specific CD8+ cells (> or = to 2 fold over pre vac value or new response detected post vacc.) | | | | | | | |
|  | Decrease in frequency of TAA-specific CD8+ cells post-vacc (< to 2 fold below pre-vacc.) | | | | | | | |
|  | Clinical responders | | | | | | | |

Abbreviations: CR: complete response; PR: partial response; SD: stable disease; PD: progressive disease; NA: not available.
